# Experimental Evidence for the Importance of Light on Understory Grass Communities in a Subtropical Forest

**DOI:** 10.3389/fpls.2020.01051

**Published:** 2020-07-10

**Authors:** Guochun Shen, Shanshan Tan, Xiaoying Sun, Yanwen Chen, Buhang Li

**Affiliations:** ^1^ Zhejiang Tiantong Forest Ecosystem National Observation and Research Station, East China Normal University, Shanghai, China; ^2^ School of Ecological and Environmental Sciences, East China Normal University, Shanghai, China; ^3^ Shanghai Institute of Pollution Control and Ecological Security, Shanghai, China; ^4^ State Key Lab of Biological Control and School of Life Sciences, Sun Yat-sen University, Guangzhou, China

**Keywords:** light gradient, abiotic filtering, biotic interactions, grass community, species composition, Bray-Curtis distance, Heishiding Nature Reserve

## Abstract

Light is one of the most important environmental filters for forest understory grass communities. It is predicted that light can select species with the same light requirements, resulting in a decrease in species compositional dissimilarity among grass communities experiencing the same light intensity, and an increase in community dissimilarity under variable light intensities. However, these predictions have been questioned recently in light of modern coexistence theories, and evidence for them in natural communities is often indistinguishable from patterns created by dispersal limitation and biotic interactions. To help fill this gap, we sampled 48 understory grass communities that had regenerated from the same soil seed bank in Southern China. Plots were established under a light intensity gradient. Changes in species composition and neighborhood densities were monitored over a growing season. Our experimental setup controls for bias from dispersal limitation and is useful for detecting the effects of biotic interactions at different intensities of light. As expected, (1) compositional dissimilarity of grass communities increased between communities with different light intensities. The extent to which communities became more dissimilar was positively correlated with the difference in the light intensity. (2) No significant change in compositional dissimilarity was observed among communities experiencing the same light intensity. (3) Finally, relative neighborhood density significantly decreased in communities with moderate to high shading treatments. Our results clearly show that light can drive compositional divergence among communities under different light densities. However, the light may not lead to convergence among communities experiencing the same low light intensity, because intense competition induced by low light might enlarge species compositional differences, as shown with the neighborhood density analysis. Therefore, our study provides more convincing evidence for the importance of light on understory grass communities in subtropical forests and highlights the need to jointly consider biotic interactions when testing for evidence for environmental filtering.

## Introduction

One of the most pervasive concepts in community ecology is the metaphor of environmental filter, which refers to abiotic factors that prevent the establishment or persistence of species in a particular location (Kraft et al., 2015). This filter is expected to drive convergence and divergence of species composition under the same and different environments, respectively (HilleRisLambers et al., 2012). Thus, species composition is commonly used to inferring the importance of light in natural communities (Baldeck et al., 2013). Light can act as an environmental filter that culling species unable to tolerate shading or intense light. It has been considered as one of the most critical forces determining community structures of forest understory plants (Ricard et al., 2003; Patry et al., 2017). However, our ability to accurately assess the importance of the light from species composition of natural forest communities has been challenged recently because some other confounding processes (e.g., dispersal limitation and biotic interactions) can produce similar community structures (Cavender-Bares et al., 2009; Mayfield and Levine, 2010). For example, low light in a shading site can lead to species compositional convergence, while similar compositional convergence may result from the inability of some species to reach the site due to dispersal limitation or the death of inferior competitors with limited light (Kraft et al., 2015). Consequently, previous evidence of light based on species composition of forest understory plants may be biased by these confounding factors, and more solid evidence on the filter effect of light is needed to better understand and protect the understory plant communities.

Two distinct methods were proposed for rigorously testing the effects of light on plant communities. The first advocates for the use of experimental manipulations that control for the different confounding processes influencing community assembly (Kraft et al., 2015). For instance, communities without interactions (e.g., individuals planted far away) and communities germinating from the same soil seed pool can be used to decouple the effects of biotic interactions and dispersal limitation from the light (Kraft et al., 2015). However, not all confounding processes can be clearly separated from light (Cadotte and Tucker, 2017a). Biotic interactions (e.g., competition and facilitation) among plants are commonplace and likely interact with light in important ways (Odling-Smee et al., 2003; Thakur and Wright, 2017). For example, competition between plants can alter light conditions beneath them, and thus these new light conditions will inevitably modify the effect of light on the small plants. In turn, these changes in light conditions can further adjust plant competition, leading to a more asymmetric light competition between plants with different heights (Hautier et al., 2009; Inman-Narahari et al., 2016). Experiments attempting to separate biotic interactions and environmental filtering of light will inevitably change the strength of both processes (Aguilar-Trigueros et al., 2017). Thus, there may be unwanted biases in the estimation of the importance of light even in well-controlled experiments.

Because of the aforementioned intrinsic linkages between light and biotic interactions, the second method advocates jointly considering both processes and detecting the effect of each process from different community structures (Cadotte and Tucker, 2017a). To accomplish this, however, more data on community structure than species compositional structures are required (Cadotte and Tucker, 2017b). For example, neighborhood density is a useful community structure since its scale dependence with distance from focal individual to neighbors can be used to detect the effect of biotic interactions (Moloney, 1993; Wiegand et al., 2017). Specifically, if abiotic environmental filtering is the only dominant force on community structures, it might alter the neighborhood density *via* death and establishment of individuals, but it does not modify the relative magnitudes of neighborhood densities at different neighborhood scales. Therefore, scale-independent changes in neighborhood density will be observed under pure environmental filtering (Møller and Waagepetersen, 2004). In contrast, if individuals interacted strongly with each other *via* biotic interactions, scale-dependent changes in neighborhood densities will be observed (Wiegand and Moloney, 2014). This is because both competition and facilitation among plants are more intense between close neighbors and are expected to generate lower and higher neighborhood densities on small spatial scales than the average neighborhood density across all examined scales, respectively (Zhu et al., 2010). Therefore, jointly considering species composition and neighborhood density provides an opportunity to understand the importance of light in the presence of biotic interactions on forest understory communities.

Here, we combine these two methods to evaluate the effects of light on understory grass communities in a subtropical forest in South China, while controlling for biases from dispersal limitation and evaluating possible influence from biotic interactions. We applied the first method by experimentally removing potential biases induced by dispersal limitation by generating grass communities from the same soil seed bank. This is in line with the second method as well, because the light can work independently of dispersal limitation after the dispersal of seeds. Next, we applied the second method to examine the effects of the light in the presence of biotic interactions. A gradient of light intensities was created using different shading treatments. If the light is important for understory grass communities, we expect that i) community compositional dissimilarity will increase among communities with distinct shading treatments, and the extent of the increase will be positively correlated with the magnitude of the difference in light intensity; ii) community compositional dissimilarity will decrease among communities experiencing the same shading treatment; and iii) changes in community compositional dissimilarities may be reduced or enhanced depending on the strength of the biotic interactions revealed by the changes of relative neighborhood densities.

## Materials and Methods

### Study Site and Experimental Design

The study was conducted within the Heishiding Nature Reserve in Guangdong Province, Southern China (23°25'15”-23°30'02” N, 111°49'09”-111°55'01” E). The reserve consists primarily of typical subtropical evergreen broadleaved forest, with a total area of 4,200 ha. The mean annual temperature of the reserve is 19.6°C, and the mean monthly temperature ranges from 10.6°C in January to 28.4°C in July. Annual precipitation is 1,740 mm, and precipitation mainly occurs between April and September. Elevation ranges from 150 to 927 m above sea level (Liang et al., 2016).

**Figure 1:**
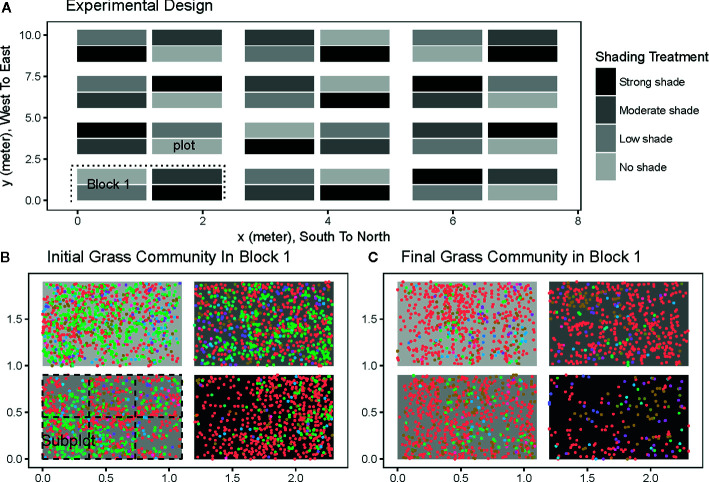
The experimental settings **(A)** for testing the light and the initial **(B)** and final **(C)** spatial distributions of grass communities in the first block treatment. The whole field in panel *a* was divided into 12 blocks (e.g., the first block in the rectangle with a dashed line around in A). Each block contains 4 plots (1.1 m × 0.9 m) with four different shading treatments: no shading (light grey), low shading (grey), moderate shading (dark grey), and strong shading (dark). Each plot was further split into 6 subplots (0.367 m × 0.3 m) to calculate inter-subplot species compositional dissimilarity (e.g., left bottom plot in B). Each colored point in panels b and c is a spatial location of an individual grass. Different point colors represent different grass species. Four abundant species are *Ageratum conyzoides* (red), *Lindernia crustacean* (green), *Cyperus rotundus* (tawny), *Lysimachia fortunei* (purple).

The experiment was conducted in an abandoned farmland that was surrounded by subtropical forest in the northwestern part of the reserve. Before the experiment, the field was dominated by *Ageratum conyzoides*, a common grass species. In March 2012, all of the grass was cut and removed; then we tilled the soil to 30 cm depth and removed large root balls from the soil. During the next 7 days, the topsoil (i.e., 0–10 cm) of the field was removed and replaced by surface soil randomly collected from the nearby forest (i.e., within 100 m away from the experimental field). The transplanted soil thus functioned as a large seed bank of mostly native understory grass species in the region. The surface soil replacement also enabled us to maximally isolate the potential bias of dispersal limitation from the light. Before transplanting, the collected soil was first homogenized by thoroughly mixing all samples. The whole field was then divided into 48 plots (**Figure 1A**). Each plot had an equal size of 1.1 × 0.9 m, and plots were placed 20 m away from the edge of the forest. All plots received 1 L/day stream water by using an automated irrigation system. Beginning in June 2012, seeds in the soil were allowed to germinate inside the plots. Seedlings outside the plots and seedlings of woody species (e.g., pioneer tree species) inside the plots were removed regularly from the field. The whole plot initially contained 47,322 individuals belonging to 39 grass species. By December 2012, plots contained 28,037 individuals belonging to 45 grass species.

### Light Intensity Gradient

Compared to other abiotic factors such as soil moisture and nutrient levels, light is the source of energy used in metabolic activities by green plants. As such, light has a large impact on the growth and development of understory plants in forest communities (Méndezdewar et al., 2015). Therefore, we created a light gradient in our experiment using four shading treatments (i.e., no shade, weak shade, moderate shade, and strong shade) (**Figure 1A**). Shading was accomplished by covering the plots from the top (i.e., 0.5 m above ground) using different black plastic shading nets. The same shading nets were wrapped around at the edges of the plots to avoid leaking light from exteriors. The illumination intensity under strong shading treatment was 10 mol·m^-2^·d^-1^), a typical illumination intensity in the understory of the subtropical forest communities in the region (Ou and Su, 2012). To minimize bias from other abiotic gradients, the entire field was divided into 12 separate blocks. Each block contained four plots exposed to the four different shading treatments (**Figure 1A**). Shading treatments were maintained until December 2012. All seedlings in each plot were identified to species, and their spatial coordinates were recorded in June 2012 and December 2012.

### Inferring the Effects of Light

The dynamics of community compositional dissimilarities (Δ*B*) among plots and subplots were used to test our first two expectations. For any two grass communities *j* and *k* we define $\Delta B_{j,k}=B_{j,k}^{t2}-B_{j,k}^{t1}$, where $B_{j,k}^{t1}$ and $B_{j,k}^{t2}$ are the Bray–Curtis dissimilarity indices between communities *j* and *k* at time *t1* and *t2*. The advantage of this index is its low sensitivity to extreme values in the data (Legendre and Legendre, 1998). A positive *ΔB*_*j*,*k*_ value indicates that communities *j* and *k* become more dissimilar to each other, and vice versa. Therefore, *ΔB*_*j*,*k*_ was tested against zero using a Student's t test to evaluate our prediction that light is important for the dynamics of community compositional dissimilarity. Regression was used to test whether there is a significant relationship between compositional dissimilarity and illumination differences among shading treatments.

### Inferring the Effect of Biotic Interactions Among Grass

The scale dependence of relative changes in neighborhood density was used to approximately detect the effect of biotic interactions on our grass communities. Here, the relative neighborhood densities of each plot were quantified using the pair correlation function, pcf(*r*), which is defined as the expected number of individuals within a ring with radius *r* centered at a specific individual. The density is then standardized by dividing by the square of the mean point density in the plot (Wiegand and Moloney, 2014). An advantage of the pair correlation function is that it is independent of the absolute density of individuals, and is unbiased from changes in the total individual density during the experiment (Møller and Waagepetersen, 2004). Thus, it can be used to compare the strength of biotic interactions at the beginning and end of the experiment, as well as among plots with different light intensities. Ripley's isotropic edge-correction method (Ripley, 1988) was used for estimating the pair correlation functions at the beginning [pcf_t1_(*r*)] and end [pcf_t2_(*r*)] of the grass communities for each plot. The effect of biotic interactions during the experiment can thus be quantified as Δpcf(*r*)=pcf_t2_(*r*)-pcf_t1_(*r*). A 95% confidence interval of Δpcf(*r*) was calculated from the 12 replicates for each shading treatment. If the whole interval of Δpcf(*r*) is below or above zero, negative (e.g., competition) and positive (e.g., facilitation) species interactions were assumed to be responsible for such changes in relative neighborhood density.

All the calculations and statistical analyses in this study were done using the R statistical software (R Core Team, 2018). The vegan R package was used to calculate species compositional dissimilarity indices and other diversity indices (Oksanen et al., 2015). The *spatstat* package was used to estimate the pair correlation function for each shading treatment (Baddeley and Turner, 2005).

## Results and Discussion

**Figure 2:**
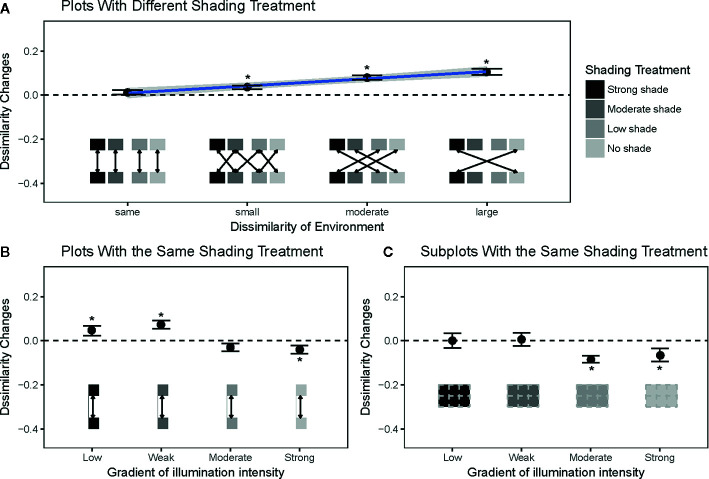
Dynamic of species compositional dissimilarity among plots with different shading treatments, along a gradient of light dissimilarity among plots **(A)**. Changes in compositional dissimilarities among plots **(B)** and subplots **(C)** with the same shading treatments. The pairs of communities to calculate the compositional dissimilarity indices are indicated by the boxes and arrows at the bottom of each panel. Different colors in the boxes represent different shading treatments. Filled points (asterisks: significant) and vertical bars are the means and one standard error bars of these changes. The horizontal dashed lines represent zero change in mean compositional dissimilarity. Solid lines and surrounded grey areas in the panel *a* were the regression line and 95% pointwise confidence interval between the change of compositional dissimilarity and illumination difference.

**Figure 3:**
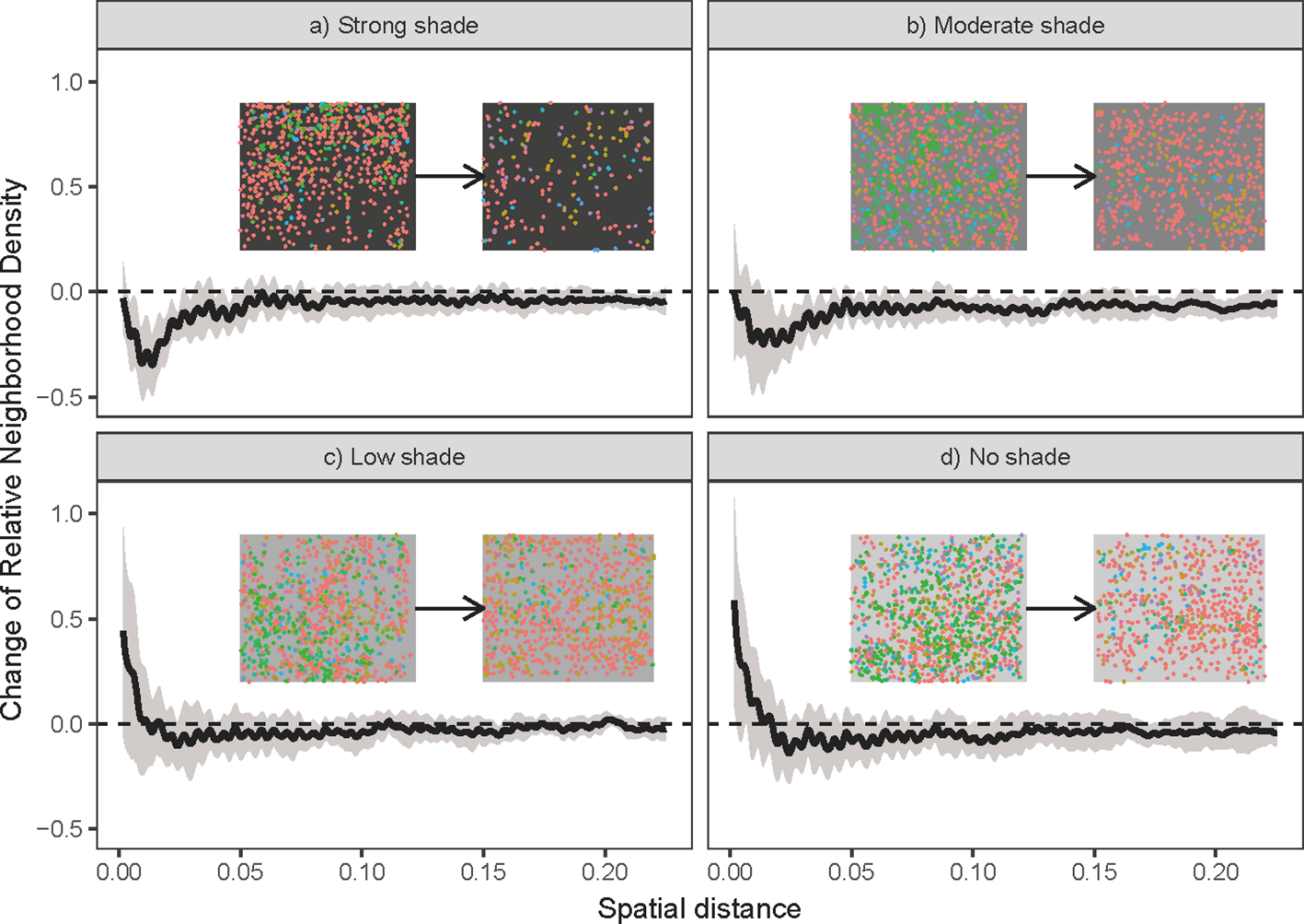
Change in the relative neighborhood density of grass communities along with spatial scales in a) strong shade, b) moderate shade, c) low shade and d) no shade treatments. The y-axis is the value of the pair correlation function of grass communities at the end of the experiment (e.g., the second grass community in the top right of each panel), minus the corresponding values of the pair correlation function of the grass community at the beginning (e.g., the first grass community in the top right of each panel). Black lines are the mean change in relative neighborhood densities across the 12 replicates for different shading treatments. Grey areas are one standard deviation confidence intervals for the mean relative density change. Horizontal dashed lines represent no change of relative neighborhood density. Points with a particular color in the top right of each panel were spatial positions of real grass individuals of a certain species (details of four abundant species and their corresponding colors are given in the legend of **Figure 1**). Points with different colors represent individuals of different species.

The grass communities showed dramatic changes in species composition during the six-month shading experiment. Compositional dissimilarities increased significantly among plots with different shading treatments and had more significant increases than the dissimilarities among plots with the same shading treatments (*t*=4.27; P < 0.01; **Figure 2A**). Specifically, there was a positive relationship between the difference in illumination among shading treatments and the differences in species composition (*R* ^2^ = 0.11; P < 0.01; **Figure 2A**). Contrary to our second expectation, there was no significant change in compositional dissimilarity among plots under the same shading treatments (**Figure 2A**). Our analyses show that this result was produced by opposite changes of dissimilarities in two types of plots, where community dissimilarity decreased among communities with no and low shading treatments and increased among communities with moderate and strong shading treatments (**Figures 2B, C**). The potential role of biotic interactions in plots with moderate to strong shading treatments was also supported by the observed changes in neighborhood density. The relative neighborhood density of grass communities at 5 cm spatial scales was reduced in both moderate and strong shading treatments (**Figures 3A, B**), and had no significant change in no to low shading treatment (**Figures 3C, D**).

By adopting the two methods of testing the environmental filtering hypothesis (Kraft et al., 2015; Cadotte and Tucker, 2017b), we provide more convincing evidence for the importance of light in understory grassland communities. Our results indicate that different light intensities can select for different species from the soil seed pool, which is consistent with our first expectation outlined in the *Introduction*. The significant increase in species compositional dissimilarity was strongest among grass communities that had the most different shading treatments. This result was independent of potential biases from dispersal limitation and suggested that light gradients can act as a filter by strongly culling understory grass species that are unable to tolerate specific abiotic conditions (Keddy, 1992; Ricard et al., 2003).

Although compositional dissimilarity increase can also arise from biotic interactions (Cavender-Bares et al., 2009), we have the following two reasons to attribute the observed increase in dissimilarity among communities with distinct shading treatments to light gradients. First, the grass communities with distinct shading treatments had a gradient of the illumination intensities since the establishment of treatments. Except for the difference of illumination intensities, all plots have similar experimental treatments, such as initial species composition and individual density. Therefore, light differences are the most likely underlying drivers of the observed increase in compositional dissimilarities among communities with distinct shading treatments. Second, even if part of the observed changes in dissimilarity among different shading treatments was caused by the different strengths of biotic interactions that emerged in the later stage of the experiment, these different biotic interactions were ultimately induced by distinct shading treatments. Specifically, shading treatments in our experiment reduced light intensities and thereby may result in the observed changes in biotic interactions (Hautier et al., 2009). For example, intensifying interspecific competition for light could be a reason. Indeed, our results suggest that interspecific competition within moderate to strong shading treatments are stronger than those in the no to low shading treatments (**Figure 3**). Importantly, the different strengths of biotic interactions may cause the observed increase in dissimilarities among distinct shading treatments, but results in **Figure 3** hinted that these different biotic interactions were most likely induced by light differences caused by distinct shading treatments. Therefore, light is an important ecological process for understanding the structure and dynamics of understory forest communities (Augspurger and Kelly, 1984; Rüger et al., 2009; Méndezdewar et al., 2015).

Our results also suggest that compositional dissimilarity may not decrease among communities with the same abiotic environment, even though it is a common expectation of the environmental filtering hypothesis (Kraft et al., 2015). Two possible reasons may falsify this expectation. First, if the strength of the light is weaker than other ecological processes (e.g., competition), community dissimilarities will not necessarily decrease among communities under the same abiotic environment. In our experiment, low light intensity within moderate to strong shading treatments may have weaker filtering forces than high light intensity on our grass communities, because species in these understory communities might have been selected by low light conditions beneath the forest canopy. This idea was supported by the relatively lower dynamic changes in the grass communities in the moderate to strong shading treatments (**Figure S1**), in which the extent of changes in Shannon-Wiener diversity, Simpson diversity, and Pielou's evenness indices of all plots was lowest in the moderate to strong shading treatments. Furthermore, the changes in relative neighborhood densities (**Figure 3**) indicate that biotic interactions (especially competition among grasses) are stronger in moderate to strong shading treatments than in no to low shading treatments. Therefore, high shading treatments will increase light competition among species, and this high-intensity competition could be strong enough to mask the effects of abiotic filtering by low light in the moderate to strong shading treatments.

The next major reason that compositional dissimilarity may not decrease under the same light conditions is that no two communities in nature can have the same abiotic environment. In our field experiment, grass communities with the same shading treatments only have very similar light intensities, yet can still have lots of small difference in other abiotic conditions such as soil nutrients and water content. These small abiotic differences may prevent convergence in community compositions, even when the same shading treatment is used. Furthermore, biotic interactions between plants and soil microbes, as well as among plants, can alter abiotic conditions (e.g., light condition at different heights) (Kraft et al., 2015). Thus, the local scale abiotic difference among communities within the same shading treatment may be amplified during the growing season. While on scales much larger than neighborhood interactions (e.g., regional scales), vast abiotic differences (e.g., mean annual temperature and precipitation) can result in powerful selection forces on species (Duivenvoorden et al., 2002). Thus, the impact of biotic interactions on community structure might be relatively low at large scales (but see Zhang et al., 2015).

Our results highlight the importance of considering biotic interactions in studies of abiotic filtering, particularly at local scales. Biotic interactions may intensify the effects of abiotic filtering among different abiotic conditions, and they can also weaken the abiotic filter under similar abiotic environments. Therefore, abiotic filtering and biotic interactions are not independent ecological processes but are internally related to each other at local scales (Cadotte and Tucker, 2017b). Any effort to fully understand the role of abiotic filtering in natural communities must simultaneously consider the effect of biotic interactions (Aguilar-Trigueros et al., 2017; Thakur and Wright, 2017). Jointly considering these two processes requires more informative community data to detect the effects of each process. Our study presents a general framework for how to detect both effects based on their distinct signatures on the neighborhood density of grasses. Similar methods can then be applied in natural communities (Zhu et al., 2010), although it will certainly be more difficult in light of complex environmental differences and potential biases from dispersal limitation and in the full context of age classes of grasses and other understory plants. Besides, community structures based on functional traits are also promised ways to study assembly rules (McGill et al., 2006). The current study only focuses on species composition and neighborhood density of recently germinated seedlings, whether the framework proposed here still works from the trait-based assembly framework in the long-term dynamic of communities remain unclear (Thakur and Wright, 2017). Finally, the experimental design does not account for a non-uniform spectral distribution of light reaching the understory plants in forests. This leads to the possibility that our shading treatments may not accurately describe the actual understory light environment, which can affect the generality of our experimental results to some extent.

In summary, our experiment shows that the species composition of understory grass communities will diverge under different intensities of light, but that they will not always converge in communities experiencing the same light intensities. This inconsistency may be attributed to the expectation that shading intensifies competition among grass species. Therefore, jointly considering both abiotic filtering and biotic interactions is necessary to fully understand and precisely project understory gass communities at local scales and under future climate change scenarios.

## Data Availability Statement

The datasets generated for this study are available on request to the corresponding author.

## Author Contributions

GS, ST, and BL designed and performed the experiment. XS, YC, and GS analyzed the data. All authors contributed to the article and approved the submitted version.

## Conflict of Interest

The authors declare that the research was conducted in the absence of any commercial or financial relationships that could be construed as a potential conflict of interest.

## Supplementary Material

Supplementary Figure 1 Appendix A. Species compositional changes during the six months of the experiment.
