# Supplementary material for: Experimental Evidence for the Importance of Light on Understory Grass Communities in a Subtropical Forest
Source: Front Plant Sci. 2020 Jul 10;11:1051. doi: 10.3389/fpls.2020.01051 (PMC7366834; doi:10.3389/fpls.2020.01051)
Supplement: Supplementary Figure 1 — Appendix A. Species compositional changes during the six months of the experiment. [file Image_1.pdf]

SUPPORTING INFORMATION: Appendix A

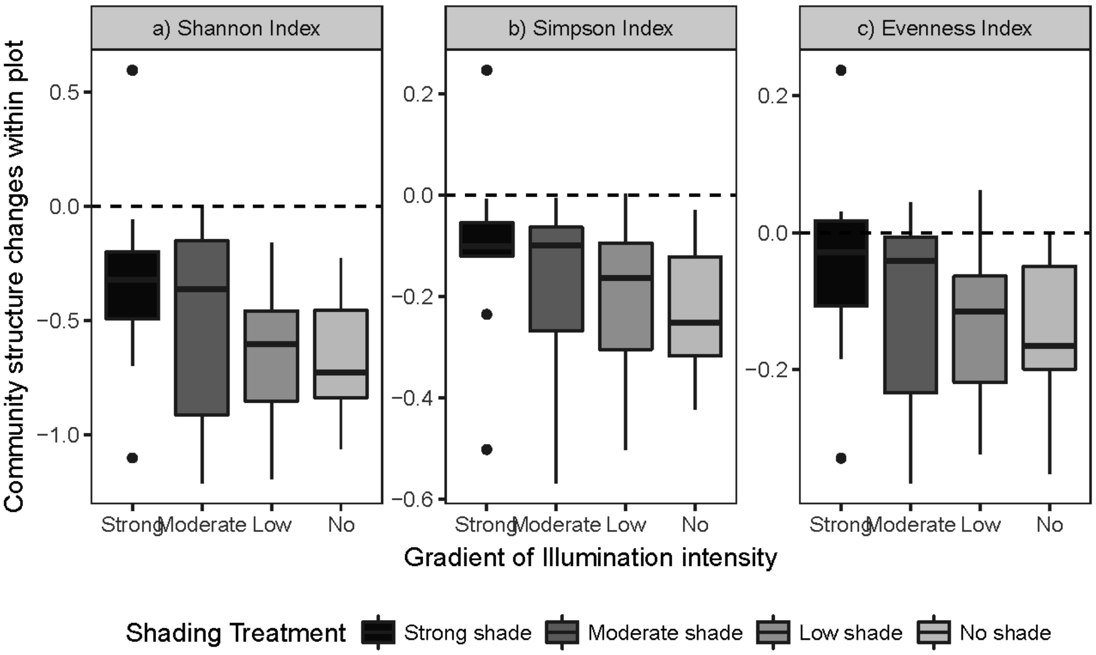

Figure S1 Boxplots of species compositional changes in the grass communities along the shading gradient. The community compositional structure was quantified using the Shannon diversity index (panel *a*), Simpson's diversity index (panel *b*) and Pielou's evenness index (panel *c*). The bottom and top of the box are the first and third quartiles of each index or rate of plots with the same shading treatment, and the band inside the box is the median. Whiskers extending vertically from the boxes indicate variability outside the upper and lower quartiles.
